# Interaction of H_**2**_S with Calcium Permeable Channels and Transporters

**DOI:** 10.1155/2015/323269

**Published:** 2015-05-11

**Authors:** Weihua Zhang, Changqing Xu, Guangdong Yang, Lingyun Wu, Rui Wang

**Affiliations:** ^1^Department of Pathophysiology, Harbin Medical University, Harbin, Heilongjiang 150086, China; ^2^The School of Kinesiology, Lakehead University, Thunder Bay, ON, Canada P7B 5E1; ^3^Department of Health Sciences, Lakehead University, Thunder Bay, ON, Canada P7B 5E1; ^4^Department of Biology, Lakehead University, Thunder Bay, ON, Canada P7B 5E1

## Abstract

A growing amount of evidence has suggested that hydrogen sulfide (H_2_S), as a gasotransmitter, is involved in intensive physiological and pathological processes. More and more research groups have found that H_2_S mediates diverse cellular biological functions related to regulating intracellular calcium concentration. These groups have demonstrated the reciprocal interaction between H_2_S and calcium ion channels and transporters, such as L-type calcium channels (LTCC), T-type calcium channels (TTCC), sodium/calcium exchangers (NCX), transient receptor potential (TRP) channels, *β*-adrenergic receptors, and N-methyl-D-aspartate receptors (NMDAR) in different cells. However, the understanding of the molecular targets and mechanisms is incomplete. Recently, some research groups demonstrated that H_2_S modulates the activity of calcium ion channels through protein S-sulfhydration and polysulfide reactions. In this review, we elucidate that H_2_S controls intracellular calcium homeostasis and the underlying mechanisms.

## 1. Introduction

Hydrogen sulfide (H_2_S) was thought for hundreds of years to be a toxic gas that smelled like rotten eggs, but the gas is now believed to be a molecule involved in intensive physiological and pathological processes [1], such as protecting the heart against acute myocardial infarction [2, 3] and ischemia/hypoxia injury, regulating blood pressure [4], mediating smooth-muscle relaxation [5], and inhibiting insulin release and renin activity [6, 7]. H_2_S, as an endogenous gasotransmitter, can be mainly generated by pyridoxal-5′-phosphate- (PLP-) dependent cystathionine *β*-synthase (CBS) and cystathionine *γ*-lyase (CSE), which interconvents the sulfuration from intracellular L-methionine and L-cysteine to produce H_2_S [8]. In addition, 3-mercaptopyruvate sulfurtransferase (3-MST) and cysteine aminotransferase (CAT) produce H_2_S from cysteine through the combined actions of both enzymes [9].

An increasing amount of evidence has demonstrated that H_2_S regulates cellular biological signaling through modulating calcium ion channels and their related transporters [10, 11], such as L-type calcium channels (LTCC), T-type calcium channels (TTCC), sodium/calcium exchangers (NCX), transient receptor potential (TRP), *β*-adrenergic receptors, and NMDA receptors. This review presents the current research on H_2_S to better understand its regulation of calcium channels, with a special emphasis on mechanisms.

## 2. The Regulatory Mechanism of H_2_S Interacting with Calcium ion Channels

### 2.1. Voltage-Dependent Calcium Channels (VDCC)

Ca^2+^ serves as an important second messenger in both excitable and nonexcitable cells. Voltage-dependent calcium channels (VDCC), store-operated calcium channels (SOCs), and G-protein coupled receptors (GPCRs) are responsible for calcium influx from extracellular fluids. Alterations in intracellular calcium levels trigger physiological responses, including cardiac muscle contraction, vascular dilatation, hormone secretion, and neurotransmitter release [12–16].

The family of VDCCs includes L-, T-, N-, and P/Q-subtypes, which differ in their cellular and subcellular distributions and functional properties [17, 18]. For example, T-type calcium channels (TTCCs) are involved in regulating cellular excitability [19], N and P/Q type channels mediate fast evoked neurotransmitter release [14], and L-type calcium channels (LTCCs) mediate excitation-contraction coupling in the heart and muscles, insulin secretion, and calcium-dependent gene transcription [20].

**Figure 1:**
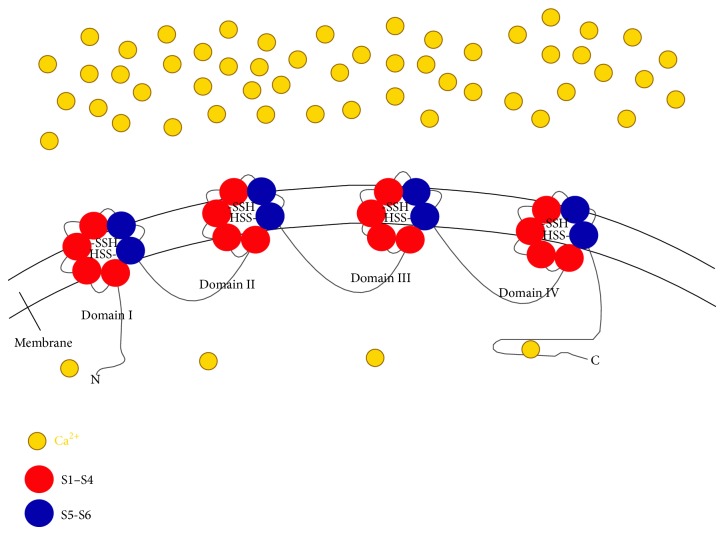
Hydrogen sulfide regulating L-type calcium channels by S-sulfhydration. LTCC consists of a pore-forming *α* subunit which contains four homologous domains (I–IV), each with six transmembrane segments (S1–S6). The S1–S4 segments are the voltage sensor, and the S5-S6 segments form the channel pore and selectivity filter. The cartoon demonstrated that H_2_S modifies the –SH from sulfhydryl donor which is transformed to free cysteine sulfhydryl and forms covalent persulfide (–SSH).

LTCCs are integral in excitation/contraction coupling and are one of the main channels for extracellular Ca^2+^ influx in myocardial cells. In 2002, Zhao and Wang first reported that H_2_S could directly inhibit calcium influx from LTCCs in smooth-muscle cells [21]. Moreover, in 2009, Sun et al. further demonstrated that H_2_S, as a novel inhibitor of LTCC, has negative inotropic effects in rat cardiomyocytes [22]. In a recent study, Avanzato et al. investigated the role of H_2_S in regulating VDCCs and the related functional effects on the cardiomyoblast cell line H9c2. They found that H_2_S inhibits LTCCs and TTCCs in H9c2. Pretreatment with NaHS (a donor of H_2_S) prevented cell death via H_2_O_2_ through inhibiting LTCCs. Their results were the first to demonstrate that H_2_S protects rat cardiomyoblasts against oxidative stress through inhibition of LTCCs [23]. In addition, Tang et al. suggested that exogenous and endogenous H_2_S inhibited pancreatic insulin secretion by inhibiting LTCCs activity. They confirmed that NaHS reversibly decreased LTCC current density in a concentration-dependent manner in CSE WT pancreatic beta cells. Furthermore, they observed that DL-propargylglycine (an inhibitor of CSE) increased the basal LTCC activity in beta cells from CSE WT mice, but not in pancreatic beta cells from CSE-KO mice. Pancreatic beta cells from CSE-KO mice displayed a higher LTCCs density than those from WT mice. These results suggested that a novel mechanism for regulating insulin secretion was related to the CSE/H_2_S system, which controlled LTCC activity [24]. Recently, some data showed that exogenous and endogenous H_2_S can modify cystein residues of different proteins through S-sulfhydration. The –SH from sulfhydryl donor is transformed to free cysteine sulfhydryl and forms covalent persulfide (–SSH) [25, 26]. In 2012, Zhang and his coworkers showed that NaHS inhibited the peak amplitude of the L-type calcium current in a concentration-dependent manner and could be partly inhibited by the oxidant sulfhydryl modifier diamide (DM). They explained that dithiothreitol (DTT), a reductant that transforms disulfide bridges into sulfhydryl groups in cysteine-containing proteins, could significantly reverse NaHS-induced inhibition of calcium current from LTCCs. Their results suggested that H_2_S inhibited L-type calcium currents depending on the sulfhydryl group in rat cardiomyocytes [27] (Figure 1).

TTCCs are classified into three T-type channel subtypes, Cav3.1, Cav3.2, and Cav3.3. There have been reports about the T-type channels being activated by H_2_S in neurons [28–30]. In the pain pathways, Cav3.2 in the peripheral terminals of nociceptors and dorsal horn spinal neurons appears to promote peripheral nociception and central nociceptive sensitization [28]. H_2_S may function as a neuromodulator in sensory transmission. There is evidence that chemotherapy-induced neuropathic pain is blocked by ethosuximide, which is known to block TTCCs. Systemic administration of dl-propargylglycine and *β*-cyanoalanine, irreversible and reversible inhibitors of CSE, respectively, also abolished neuropathic pain. Okubo et al. demonstrated that Ca_v_3.2 and CSE at the protein level are upregulated, which induced a significant increase in H_2_S level. H_2_S facilitated pain sensation by targeting Ca_v_3.2 TTCCs. The H_2_S/Ca_v_3.2 pathway appears to play a role in the maintenance of surgically evoked neuropathic pain [31]. Intraplantar administration of NaHS causes mechanical hyperalgesia in rats, an effect reversed by mibefradil (a T-type Ca^2+^ channel blocker), and also enhances membrane currents through the TTCC in NG 108-15 cells and mouse dorsal root ganglion neurons [29, 30]. Their data suggested that spinal and peripheral NaHS/H_2_S facilitates the expression of Cav3.2 TTCCs in the primary afferent and/or spinal nociceptive neurons, leading to sensitization of nociceptive processing and hyperalgesia [31]. Sekiguchi et al. demonstrated that endogenous and exogenous hydrogen sulfide facilitate T-type calcium channel currents in Cav3.2-expressing HEK293 cells [32]. In contrast, Elies et al. reported an inhibitory effect with high doses of NaHS on Cav3.2-overexpressing HEK cells [33]. Their data were the first preliminary evidence that H_2_S negatively modulates endogenously expressed TTCCs in a myoblast cell line. In spite of the opposite opinion in the effects of NaHS on TTCCs in different research groups, H_2_S regulating the activity of TTCC has been confirmed widely. However, most of the evidence suggests that H_2_S elevates the activities of TTCCs and increases the amplitudes of T-type Ca^2+^ currents in different cell lines.

### 2.2. *β*-Adrenergic Receptors

Cardiac excitation-contraction coupling is under the direct control of the adrenergic nervous system. In the heart, the *β*-adrenergic receptor (AR), a G-protein coupled receptor, activates the associated adenylyl cyclase (AC)-cAMP-protein kinase A (PKA) pathway [34]. *β*-Adrenoceptor-coupled stimulatory G proteins lead to an increased intracellular cAMP level and stimulate protein kinase A (PKA) to mediate phosphorylation of LTCCs and finally increase contractile function [35–37]. Some reports have observed that H_2_S content in the heart was significantly reduced in a cardiac ischemia [38] and overstimulation of the *β*-adrenergic system by isoproterenol (ISO, *β*-adrenoceptor agonist) models [39]. Yong and his coworkers revealed that H_2_S may negatively modulate *β*-adrenoceptor function via inhibiting adenylyl cyclase activity [40]. They found that ISO (10^−9^–10^−6 ^M), in a concentration-dependent manner, increased the twitch amplitude of ventricular myocytes, which was attenuated by NaHS (10^−5^–10^−3 ^M) in a dose-dependent manner. The amplitudes and maximal velocities (±dL/dt) for myocyte twitch and EI-[Ca^2+^]_i_ transient amplitudes were enhanced by ISO, forskolin (an adenylyl cyclase activator), 8-bromoadenosine-3′,5′-cyclic monophosphate (an activator of protein kinase A), and Bay K-8644 (a selective LTCC agonist). Administration of NaHS (100 *μ*M) significantly attenuated the effects of only ISO and forskolin. Moreover, NaHS reversed the ISO-induced cAMP increase and forskolin-stimulated adenylyl cyclase activity. Thus, they postulated that H_2_S may negatively regulate *β*-AR function through inhibition of the cAMP/PKA pathway. In addition, some studies found that the plasma concentration of H_2_S in patients with coronary heart disease [41] and in the setting of ISO overstimulation significantly decreased endogenous H_2_S production, which implies that a reduced H_2_S level caused by ischemia and *β*-adrenoceptor overstimulation may result in impairment of the negative modulation of H_2_S on the *β*-adrenoceptor system and hence calcium overload.

### 2.3. Sodium Calcium Exchanger (NCX)

The sodium calcium exchanger (NCX) is one of the key players in the regulation of intracellular calcium homeostasis. In a physiological condition, NCX, a nonselective cation channel, may induce the influx of 3 Na^+^ into cells in exchange for the efflux of 1 Ca^2+^ [42]. However, in pathological conditions, such as ischemia/reperfusion, hypoxia, and heart failure, NCX function could be reversed, with one Ca^2+^ moving inward and three molecules of Na^+^ going out of the cell [43]. H_2_S may stimulate Ca^2+^ influx into endothelial cells (ECs) by recruiting the reverse-mode for the NCX [44–46]. To confirm the role of NCX in NaHS-dependent Ca^2+^ signaling, KB-R 7943 (20 *μ*M), a selective inhibitor of the reverse-mode, was used in the experiment. Moccia and his coworkers' data showed that NaHS failed to elicit a [Ca^2+^]_i_ elevation in ECs pretreated with KB-R 7943. In addition, the amplitude of the Ca^2+^ response was significantly lower in ECs activated by the H_2_S donor in the presence of KB-R 7943. Taken together, these findings hinted at NCX as a key mediator of NaHS-elicited Ca^2+^ inflow in rat aortic ECs. To further determine the effect of sulfide signaling on the NCX, several studies investigated NCX expression and function in HeLa cells. They observed increased levels of NCX1 mRNA, protein, and activity after 24 h of GYY4137 (morpholin-4-ium-4-methoxyphenyl(morpholino) phosphinodithioate, a slow releasing H_2_S donor) treatment. This increase was accompanied by elevated cAMP due to GYY4137 treatment, which was completely abolished when NCX1 was silenced. An increased cAMP level would point to upregulation of the *β*-adrenergic receptors. Thus, Cheng et al. investigated the relationship of *β*-adrenergic receptors with the NCX1 in the presence and/or absence of H_2_S and determined the physiological importance of this potential communication using GYY4137 [47]. Indeed, GYY4137 increased expression of the *β*1 and *β*3 (but not *β*2) adrenergic receptors, suggesting that sulfide signaling played a role in regulating the NCX1 and *β*1 and *β*3 adrenergic receptors and their colocalization.

### 2.4. Transient Receptor Potential (TRP) Channels

A growing body evidence has shown that H_2_S and neuronal excitation induce calcium ion influx in astrocytes, and the interaction between neurons and astrocytes regulates synaptic activity [48–50]. TRP channels were found to mediate the responses to H_2_S in the urinary bladder and sensory neurons [51]. Although the effects of H_2_S on transient receptor potential (TRP) channels are not completely clear, Kimura et al. demonstrated that polysulfides of H_2_S-derived signaling molecules stimulated TRP channels in the brain [52]. They suggested that H_2_S induced Ca^2+^ influx in astrocytes through generating polysulfides of TRP. They administered sodium polysulfides, Na_2_S_3_, in their experiments, which induced Ca^2+^ influx in a concentration-dependent manner. They also confirmed that this astrocyte response to H_2_S was suppressed by the TRP channel blockers La^3+^ and Gd^3+^. To further reveal the mechanism for Na_2_S_3_-induced TRP channel opening, the TRPA1 channel inhibitors HC-030031 and AP-18 and TRPA1 siRNA were used. Their data showed that, in the presence of the inhibitors or TRPA1 siRNA, Na_2_S_3_ could not induce Ca^2+^ influx through the TRPA1 channel. Liu et al. showed that H_2_S maintained mesenchymal stem cell function via regulation of Ca^2+^ channel sulfhydration [53]. They found that NaHS-treated bone marrow mesenchymal stem cells (BMMSCs) induced Ca^2+^ influx with a limited contribution from intracellular Ca^2+^ storage. They also found that DTT, by reducing the disulfide bonds in proteins and increasing the number of residual sulfhydryl proteins, elevated NaHS-induced Ca^2+^ influx in BMMSCs. Diamide, by reducing the number of sulfhydryls and 2-sulfonatoe-methanethiosulfonate (MTSES), a nonpermeable reagent able to reduce free sulfhydryls only on the outer cytomembrane, could reduce NaHS-induced Ca^2+^ influx in BMMSCs. These results revealed that free sulfhydryls affect NaHS-induced Ca^2+^ influx. The above results suggested that polysulfides, as H_2_S-derived bioactive molecules, stimulate TRP channels, providing a new molecular mechanism for sulfide-induced signaling.

### 2.5. N-Methyl-D-aspartate Receptors (NMDARs)

N-Methyl-D-aspartate receptors (NMDARs) form glutamate-gated ion channels that are widely expressed in the central nervous system and are highly permeable to calcium ions, which are essential for regulating synaptogenesis, use-dependent synaptic remodeling, and long-term plastic changes in synaptic strength [54]. H_2_S, as a neuromodulator, elevates the activity of N-methyl-D-aspartate (NMDA) receptors to facilitate the induction of hippocampal long-term potentiation (LTP), a synaptic model of memory formation [48, 55]. Nagai et al. demonstrated that H_2_S enhances the neuronal response to glutamate and induces Ca^2+^ waves in astrocytes [49]. Glial cells communicate with surrounding cells by increasing the intracellular concentration of Ca^2+^ and propagating the signal as Ca^2+^ waves that occur in glia, and neurons show Ca^2+^ oscillations and intracellular Ca^2+^ waves. Because astrocytes elicit intracellular Ca^2+^ waves by electrical stimulation and application of NMDA in mixed cultures of neurons and astrocytes, astrocytes have been suggested to respond directly to a neurotransmitter released from neurons excited by NMDA or electrical stimulation [56–59]. La^2+^ and Gd^3+^ block Ca^2+^ waves and inhibit Ca^2+^ channels; La^2+^ and Gd^3+^ may inhibit the exocytosis of glutamate or some factor from neurons when neurons are stimulated by NMDA. However, La^2+^ and Gd^3+^ block H_2_S-initiated waves in pure astrocyte culture, showing that Ca^2+^ is most likely involved in the propagation step. H_2_S released in response to neuronal excitation may activate Ca^2+^ channels to induce Ca^2+^ waves in astrocytes. H_2_S may therefore mediate signals between neurons and glia. H_2_S is released from neurons or glia by neuronal excitation and increases the intracellular concentration of Ca^2+^ by activating Ca^2+^ channels in astrocytes and to a lesser extent causes release from intracellular Ca^2+^ stores. An elevated intracellular Ca^2+^ triggers the induction of Ca^2+^ waves that propagate to the neighboring astrocytes [60–63]. H_2_S enhances the activity of NMDA receptors by reducing the cysteine disulfide bond in the hinge region of the ligand-binding domain of NMDA receptors, and polysulfides further enhance this activity by adding bound sulfane sulfur to the receptors. Polysulfides activate the TRPA1 channels in astrocytes to induce Ca^2+^ influx, which facilitates the release of the gliotransmitter D-serine to enhance the activity of NMDA receptors. By these integrated mechanisms, H_2_S along with polysulfides may facilitate the induction of LTP [64].

## 3. Conclusions and Perspective

An increasing amount of evidence has clearly demonstrated that H_2_S is associated with relevant biological processes, such as cardiac systolic function, sensory transduction, antiapoptotic function, and neuroprotection [65]. These functions are closely related to H_2_S regulating various calcium ion channels and transporters [66]. Many studies cited in this review investigated the fact that polysulfides of calcium ion channels, which are modified by H_2_S, have been found to elevate the activity of TRP, TTCC, and NMDARs and to inhibit LTCC through the mechanism of sulfhydration. Furthermore, H_2_S could upregulate the activities of the NCX1 and *β*1 and *β*3 adrenergic receptors and their colocalization. Altered effects of H_2_S on calcium ion channels under different pathophysiological conditions are being investigated. Extensive research on the mechanisms of H_2_S modulation of calcium signaling will provide new insights into the physiological function of H_2_S.
